# Lag-time in Alzheimer’s disease patients: a potential plasmatic oxidative stress marker associated with ApoE4 isoform

**DOI:** 10.1186/s12979-019-0147-x

**Published:** 2019-04-01

**Authors:** Luca Massaccesi, Emanuela Galliera, Daniela Galimberti, Chiara Fenoglio, Marina Arcaro, Giancarlo Goi, Alessandra Barassi, Massimiliano Marco Corsi Romanelli

**Affiliations:** 1 Department of Biomedical Sciences for Health, Università degli Studi di Milano, Milan, Italy; 2 IRCCS Galeazzi Orthopaedic Institute, Milan, Italy; 3 Department of Pathophysiology and Transplantation, Università degli Studi di Milano, Centro “Dino Ferrari”, Milan, Italy; 4 U.O.S.D. Neurologia-Malattie Neurodegenerative, Fondazione IRCCS Ca’ Granda Ospedale Maggiore Policlinico, Milan, Italy; 5 Department of Biomedical, Surgical and Dental Sciences, Università degli Studi di Milano, Milan, Italy; 6 Department of Health’s Science, Università degli Studi di Milano, Milan, Italy; 7 U.O.C SMEL-1 Patologia Clinica IRCCS Policlinico San Donato, San Donato, Milan, Italy

**Keywords:** Oxidative stress, Vascular dysfunction, Age-associated diseases, Alzheimer’s disease

## Abstract

In the brain, Oxidative Stress (OS) contribute to structural and functional changes associated with vascular aging, such as endothelial dysfunction, extracellular matrix degradation, resulting in age-related reduced vasodilatation in response to agonists. For this reason, OS is considered a key factor in Alzheimer’s Disease (AD) development and recent evidence correlated oxidative stress with vascular lesion in the pathogenesis of AD, but the mechanism still need to be fully clarified.

The etiology of AD is still not completely understood and is influenced by several factors including Apolipoprotein E (ApoE) genotype. In particular, the Apo ε4 isoform is considered a risk factor for AD development.

This study was aimed to evaluate the possible relationship between three plasmatic OS marker and Apo ε4 carrier status. Plasmatic soluble receptor for advanced glycation end products (sRAGE) levels, plasma antioxidant total defenses (by lag-time method) and plasmatic Reactive Oxygen species (ROS) levels were evaluated in 25 AD patients and in 30 matched controls. ROS were significantly higher while plasma antioxidant total defenses and sRAGE levels were significantly lower in AD patients compared to controls. In AD patients lag-time values show a significant positive linear correlation with sRAGE levels and a (even not significant) negative correlation with ROS levels. Lag-time is significantly lower in ε4 carrier (*N* = 13) than in ε4 non-carrier (*N* = 12). Our result confirms the substantial OS in AD. Lag-time levels showed a significant positive correlation with sRAGE levels and a significant association with ε4 carrier status suggesting that plasmatic lag-time evaluation can be considered as a potential useful OS risk marker in AD.

## Introduction

Alzheimer disease (AD) accounts for the largest proportion of dementia diseases in the older population [1]. Recent evidences indicated that vascular dysfunction and damage are linked to cerebrovascular disorders in the elderly and increase significantly AD incidence [2]. The vascular endothelium is a major target of oxidative stress (OS) caused by Reactive Oxygen species (ROS), which play a critical role in the pathophysiology of vascular disease. ROS are important regulators of the inflammatory response: on one hand, at low concentration they act as regulators of cell growth and activity in the inflammatory process, on the other hand, at high concentration they have deleterious effects on cells and tissues [3]. The oxidative stress results from an imbalance between ROS and antioxidant molecules, resulting in an excess of ROS leading to cell injury and death and it is commonly associated with ageing process and age-related degenerative disorders [4]. Compared to other organs, the brain is more vulnerable to oxidative stress due to its high rate of oxygen consumption [4]. In the brain, OS also contribute to structural and functional changes associated with vascular aging, such as endothelial dysfunction, extracellular matrix degradation, resulting in age-related reduced vasodilatation in response to agonists [5]. For this reason OS is considered a key factor in AD development since asymptomatic stages [6–8]. Recent evidences correlated oxidative stress with vascular lesions in the pathogenesis of AD, but the mechanism still need to be fully clarified.

The etiology of AD is still not completely understood and is influenced by several factors including Apo lipoprotein E (ApoE) genotype [9], considered an important risk factor for AD. In particular, ApoE exists in three isoform (ApoE2, ApoE3 and ApoE4) but the risk for AD development strongly increases only in the ApoE4 (ε4) carriers [10]. The aim of this study is to measure plasmatic OS markers in AD patients to evaluate the possible role of OS biomarkers as noninvasive blood-based tool to evaluate AD diagnostic and monitoring. In addition, plasmatic OS markers will be evaluated in ApoE4 (ε4) carrier and not carrier AD patients, in order to identify a possible correlation with ApoE4 (ε4) status and define a potential profile of risk factors for AD development.

## Material and methods

### Subjects

Twenty-five patients with Alzheimer’s Disease (AD), aged 74.0 ± 6.34; including 13 ε4 carrier and 12 ε4 non-carrier, were recruited from UOSD Neurologia-Malattie Neurodegenerative, Fondazione IRCCS Ca′ Granda Ospedale Maggiore Policlinico, Milan, Italy. The control group was composed by 30 adult volunteer blood donors, aged 75.36 ± 10.71, from the Italian Association of Blood Volunteers (AVIS) in Milan, Italy. The study was carried out in accordance with recommendation of ethical committee of Fondazione IRCCS Ca′ Granda Ospedale Maggiore Policlinico (approval number: 441/2016) All subjects gave written informed consent in accordance with the Declaration of Helsinki.

### Materials

Commercial chemicals were of the highest available grade. The water routinely used was freshly redistilled in a glass apparatus. Copper (II) sulphate (CuSO_4_), was purchased from Sigma Chemical Co. (St. Louis, MO, USA). All other reagents were purchased from Merck (Darmstad, Germany). D-ROMs kit test was purchased from Diacron International (Grosseto, Italy).

### Blood samples and serum/plasma preparation

Plasma was prepared from heparinized venous blood. After collection, blood samples were immediately centrifuged for 15 min at 3000×g and plasma immediately withdrawn and stored at − 20 °C until ELISA assay and evaluation of plasmatic oxidative status.

### Evaluation of plasma oxidative status

Plasma lipid hydroperoxide levels (ROS) were determined colorimetrically according to Trotti et al. [11] and expressed as H_2_O_2_ equivalents.

The kinetics of plasma oxidation, induced by addition of CuSO_4_ 0.5 M, were determined at 37 °C by monitoring the development of fluorescence at 430 nm, setting the excitation at 355 nm as described by Cervato et al. [12] by Multilabel Counter Wallac 1420 from PerkinElmer. This method allows the evaluation of the peroxidation kinetics monitored following the formation of fluorescent adducts originating from the reaction of aldehydes (derived from lipid peroxidation promoted by Cu++ bound to apolipoproteins) with amino groups of plasma proteins and/or phospholipids. The kinetic is expressed by a sigmoid curve that can be divided into an initial latency phase, followed by a second propagation phase. The initial latency phase (lag time, expressed in minutes and calculated as the intercept of the linear regression of the propagation phase with that of the latency phase) is an index of lipoprotein resistance to peroxidation.

### sRAGE ELISA assay

Levels of soluble RAGE (Receptor of Activated Glycoslation Endproducts) in plasma were determined by ELISA commercial assays, according to the manufacturers’ instructions (sRAGE: R&D Systems, Minneapolis, Minnesota, USA). For the sRAGE assay, the sensitivity was 4.44 pg/mL, and intra- and inter-assay coefficients of variation were 2.4 and 4.7%, respectively.

### Statistical analysis

The Shapiro–Wilk test showed no significative difference from normal distribution. Therefore, parametric techniques were used. Means were compared by Student t-test. The Pearson correlation coefficient (r^2^) was calculated to determine the correlation between values measured by different assays. Distribution and correlation analysis were performed using the SPSS STATISTIC 25 package (SPSS Inc., Chicago, IL, USA).

## Results

Plasma peroxidation parameters.

**Fig. 1:**
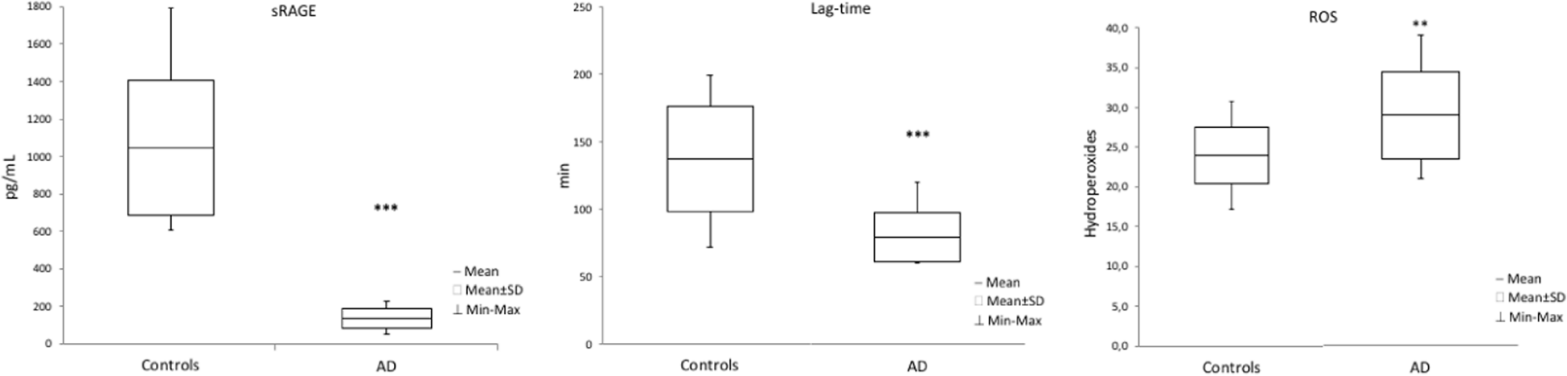
Plasma peroxidation parameters. The following parameters was measured in AD jj patients and controls: Soluble RAGe (sRAGE, pg/mL), Lag Time (min) and ROS (Hydroperoxides). Hydroperoxides are expressed as equivalent of H_2_O_2_ mg/dL of plasma. Results are expressed ad mean ± SD. ∗∗ *P* < 0.01 ∗∗∗ *P* < 0.001 controls versus AD subjects

ROS were significantly higher (*P* < 0,01) in AD patients compared to controls, while plasma antioxidant total defenses (measured by lag time) and sRAGE levels were significantly lower *(P < 0,001)* in AD patients compared to controls (Fig. 1).

**Fig. 2:**
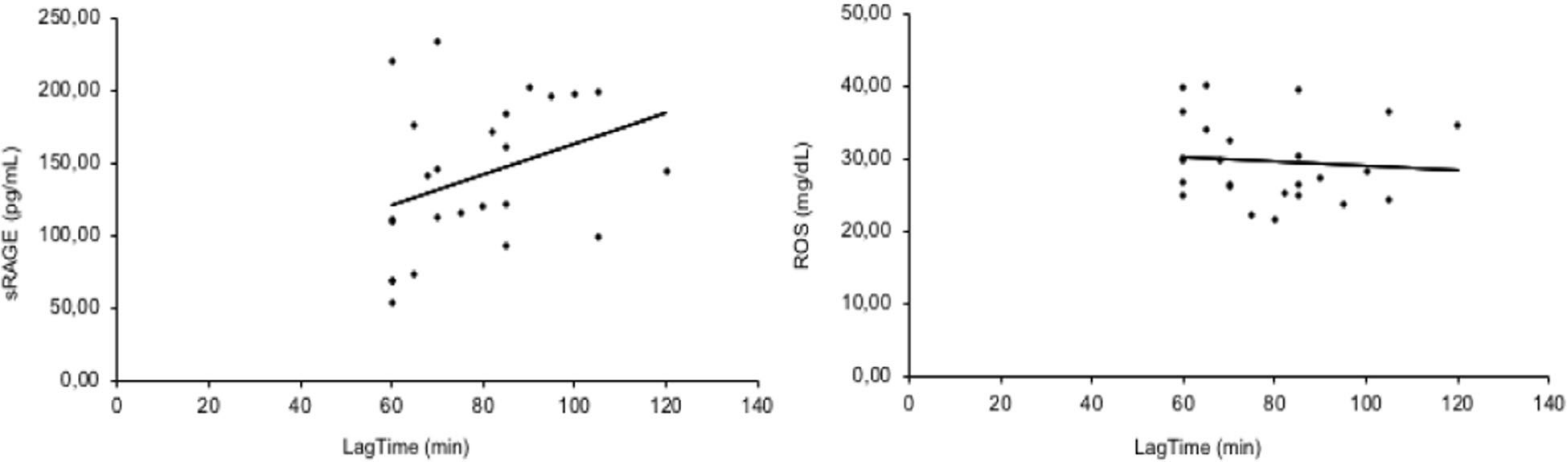
Correlation analysis between lag time and others oxidative parameters in AD patients. Lag-time values show a significant (*p* < 0,05) positive linear correlation (r^2^ = 0,347) with sRAGE levels (ng/mL) and a (even not significant) negative correlation (r^2^ = − 0,101) with ROS (mg/dL) levels

In AD patients lag-time values show a significant (*P* < 0,05) positive linear correlation with sRAGE levels and a negative correlation, even though not significant, with ROS levels (Fig. 2).

Evaluation of OS according to ApoE4 ε4 carrier status.

**Fig. 3:**
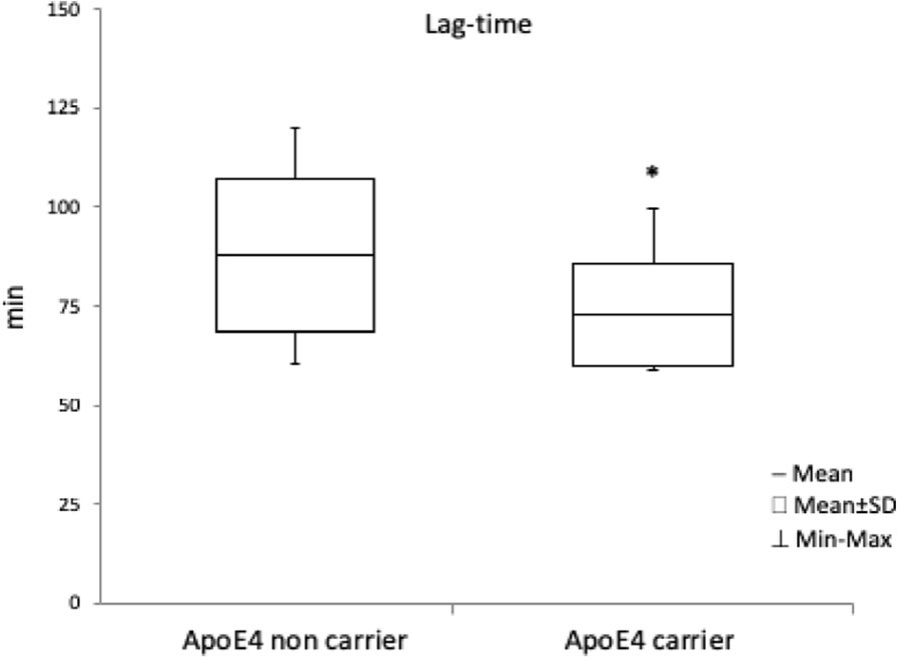
Variation of plasma peroxidation parameter in ApoE4 carrier. Variation of plasma peroxidation parameter (Lag Time) was evaluated in ApoE4 carrier (*N* = 13) compared to ApoE4 non carrier (*N* = 12). Results are expressed ad Mean ± SD ∗ 푃 < 0.05 e4 carrier versus e4 non-carrier.

Lag-time is significantly lower (*P* < 0,05) in ε4 carrier than in ε4 non-carrier (Fig. 3).

## Discussion

The brain is the most metabolically active organ in the human body and requires around the 20% of the whole organism’s energy. There are increasing evidences that a failure in the ability to maintain a well-balanced ROS level causes cerebrovascular dysfunction, which promotes an increase in blood-brain-barrier (BBB) leakage interfering with brain energy supply and homeostasis and increasing amyloid β (Aβ) peptide deposition in vascular walls [13]. Alterations in BBB features are associated with oxidative stress and inflammatory processes, leading to neurodegenerative conditions resulting in a decline of cerebrovascular function, typical of age-associated diseases, such as AD [8, 13, 14].

It was demonstrated that the risk for AD is strongly increased in the ApoE4 (ε4) carriers [10] and that the occurrence of OS in neurological disorders as mild cognitive impairment (MCI) could be related to ε4 carrier status [15].

In our study we have evaluated in AD patients, both in ApoE4 (ε4) carriers and non-carriers, three plasmatic OS markers (Plasmatic soluble receptor for advanced glycation end products – sRAGE - levels, plasma antioxidant total defenses, by lag-time method, and plasmatic ROS levels in order to evaluate a possible association between these OS markers and ApoE4 (ε4) carrier status.

To evaluate the antioxidant defenses in vivo we evaluated the total “oxidation resistance” of plasma lipoproteins using the fluorescent kinetics method of Lag-time [12, 16] that provides a comprehensive picture of plasma susceptibility to peroxidation in comparison with the conventional measurements of antioxidant and pro-oxidant ratios, including the antioxidant enzyme activities [17].

Several studies in literature reports showed an increase of OS markers in AD patients, confirming the strong relation between OS and AD [5, 18–20]. In addition, the presence of oxidative stress markers such as advanced glycation End Product (AGE) has been detected in AD patients [4, 21, 22]. Consistently, we observed a significative increase of ROS in AD patients, associated with a significative decrease in antioxidant defense, measured by lag time and significative decrease of the soluble AGE receptor sRAGE. sRAGE have been extensively studied as protective factor against AGE induced cell damage [23–26]. Indeed, soluble receptor for AGE (sRAGE) counteracts the adverse effects of AGE-RAGE interaction by competing with RAGE for binding with AGE and low levels of serum sRAGE have been proposed as a biomarker for diseases, including AD [25]. In our study, sRAGE also showed a significative correlation with the other two biomarkers of oxidative stress, lag time and ROS, confirming that in AD patient’s sRAGE can be considered a useful tool to evaluate OS status, along with ROS and lag time. Many end-products of peroxidation have been identified in the brain tissue and in blood circulation of AD patients suggesting that OS biomarkers could represent useful tools for noninvasive blood-based AD diagnostic and monitoring. Considering that the ideal biomarker should be not only rapid, reproducible, easy to perform but also less invasive as possible, in particular in the elderly, the plasmatic OS determination in AD clinical practice is getting an increasing importance [27, 28]. A first correlation between carrier and OS biomarkers in neurodegenerative disease was suggested in mild cognitive impairment (MCI) where a positive correlation was observed between superoxide dismutase activity and MCI [29], and in the neurodegenerative aspects of AD [30, 31]. Previous evidences showed a specific pattern, characterized by higher tHcy and lower TAC levels, was observed in AD, indicating that oxidative imbalance seems to play a role in the pathogenesis of AD [32]. In addition, Miyata and Smith [33] showed that ApoE has allele-specific beneficial effect against free radicals reducing neuronal death due to hydrogen peroxide and beta-amyloid through antioxidant activity. This protective effect is detectable with the E2 isoform but tends to vanish with E3 isoform and almost disappear with E4 isoform, indicating that the ApoE4 (ε4) isoform lack the antioxidant protective activity and is more prone to OS damage [34, 35].

According to all these evidences our data showed a consistent OS condition in the AD patients with an intriguing result regarding plasma antioxidant total defenses (lag-time).

Lag-time method allows a complete evaluation of the total plasmatic antioxidant defenses in vivo in comparison with the conventional measurements of pro-oxidant markers. Indeed, despite a practically equivalent OS levels in e4 carrier and not carrier (as pointed out from ROSs and sRage values), lag-time results to be significantly lower in the e4 carrier subject. Indeed, this parameter resulted significantly lower in the ApoE4 (ε4) carriers in comparison to the not carrier suggesting a significant decrease of antioxidant defenses in these subjects, consistently with a previous work in a murine model by Myata and Smith showing, in a murine model, the decrease of the antioxidants activities due to the presence of the allele ApoE4 [33] (ε4).

Taken together these results indicated that Lag-time levels, as measure of antioxidant defenses, shown a significant decrease in APOε4 carrier and a positive correlation with the decrease of the protective factor sRAGE levels.

## Conclusion

Our results confirm the substantial OS in AD. Lag-time levels, as measure of antioxidant defenses, showed a positive correlation with ApoE4 (ε4) carrier status and sRAGE, suggesting that plasmatic lag-time evaluation can be considered as a useful OS marker for monitoring AD patients and as a further potential risk marker for AD development.

## Abbreviations

AD: Alzheimer’s Disease
ApoE: Apolipoprotein E
OS: Oxidative Stress
ROS: Reactive Oxygen Species
sRAGE: soluble Receptor for Advanced Glycation End-products
